# Maize seed appearance quality assessment based on improved Inception-ResNet

**DOI:** 10.3389/fpls.2023.1249989

**Published:** 2023-08-24

**Authors:** Chang Song, Bo Peng, Huanyue Wang, Yuhong Zhou, Lei Sun, Xuesong Suo, Xiaofei Fan

**Affiliations:** College of Mechanical and Electrical Engineering, Hebei Agricultural University, Baoding, China

**Keywords:** corn seed, quality assessment, depthwise separable convolution, attention mechanism, feature fusion

## Abstract

Current inspections of seed appearance quality are mainly performed manually, which is time-consuming, tedious, and subjective, and creates difficulties in meeting the needs of practical applications. For rapid and accurate identification of seeds based on appearance quality, this study proposed a seed-quality evaluation method that used an improved Inception-ResNet network with corn seeds of different qualities. First, images of multiple corn seeds were segmented to build a single seed image database. Second, the standard convolution of the Inception-ResNet module was replaced by a depthwise separable convolution to reduce the number of model parameters and computational complexity of the network. In addition, an attention mechanism was applied to improve the feature learning performance of the network model and extract the best image information to express the appearance quality. Finally, the feature fusion strategy was used to fuse the feature information at different levels to prevent the loss of important information. The results showed that the proposed method had decent comprehensive performance in detection of corn seed appearance quality, with an average of 96.03% for detection accuracy, 96.27% for precision, 96.03% for recall rate, 96.15% for F1 value of reconciliation, and the average detection time of an image was about 2.44 seconds. This study realized rapid nondestructive detection of seeds and provided a theoretical basis and technical support for construction of intelligent seed sorting equipment.

## 1 Introduction

Maize is an essential cereal crop that is widely grown worldwide and has an increasing production and trade volume (Ali et al., 2020). Appearance quality is an important factor affecting the price of corn seeds, and effective identification of seed quality is critical for ensuring food security and agricultural production safety. With the rapid advancements in automation, machine vision technology (Huang et al., 2019; Kim et al., 2020; Wang and Xiao, 2020; Ansari et al., 2021; Lu et al., 2022) can be used to nondestructively and quickly obtain seed surface feature information at a low cost and high detection accuracy and efficiency, thereby providing potential new methods for seed quality identification.

Machine learning (Rahmani et al., 2016; Prajapati et al., 2018; Liang et al., 2019; Sharaff et al., 2021) is an active field of artificial intelligence research that has advantages in terms of training small data samples and wide applications in agricultural product identification and defect detection. Farooqui et al. (2019) used a gray-level co-occurrence matrix for disease feature extraction, a support vector machine classifier for plant disease identification, combined with advanced neural network to optimize the data to improve the detection accuracy and demonstrated the feasibility of this approach for plant disease diagnosis through experiments. Effective classification of seeds is an important part of selecting and breeding good seeds. To simplify the seed selection process, Ma et al. (2021) proposed a peanut seed appearance quality detection method, using peanut seed size and appearance color as the main features and a support vector machine classification model to complete the classification task. The experimental results showed that the method had an accuracy of 86% for the classification of bulk peanut seeds, which met the preliminary classification requirements of peanut seeds in actual production. Gao et al. (2016) designed a fresh corn quality detection classifier, which analyzed the texture features of fresh corn images by wavelet analysis method, used the maximum entropy function to measure the separation degree of the texture images, and combined with the weight criterion to classify the fresh corn of different varieties, sizes, and damage degrees, and the effective classification rate could reach more than 99%. Zhao et al. (2022) extracted three categories of raw coffee bean features: contour, color, and texture to detect defective raw coffee beans by the features of a single category or category combinations. The findings were applied to a grid search to determine support vector machine classification model parameters and combined with a k-fold cross-validation test to compare support vector machine model performances. The experimental results showed that the average accuracy, precision, recall, and F1 values were 84.9%, 85.8%, 82.3%, and 84.0%, respectively. This method provided a theoretical base for the automatic detection of defective raw coffee beans. With the requirements of strict and precise agricultural development, there is an urgent need to explore new research methods to achieve precise assessment of seed appearance quality and promote intelligent agricultural development.

With the rapid development of deep learning, convolutional neural networks are widely used in the fields of medicine, aviation, and agriculture because of their excellent feature learning and expression capabilities (Kamilaris and Prenafeta-Boldu, 2018; Naranjo-Torres et al.,2020; Zhang et al., 2020; Cong and Zhou, 2022; Liu et al., 2023). Compared with traditional machine learning techniques, convolutional neural networks are more generalizable, faster to train, and can obtain significant information directly from images, which eliminates the tedious steps of manually extracting image features used in traditional methods. In applications for agriculture, convolutional neural networks are often used in areas such as the classification of crop pests and diseases (Wu et al., 2019; Peng et al., 2019; Tiwari et al., 2021; Liu et al., 2022; Liu et al., 2022), agricultural product species identification (Ajit et al., 2020; Gao et al., 2020; Chen et al., 2021; Laabassi et al., 2021; Sj et al.,2021), yield estimation (Zhang et al., 2020; Tan et al., 2019; Alexandros et al., 2023; Kavita et al., 2023), and crop quality grading (Anikó and Miklós, 2022; Liu et al., 2022; Li et al., 2022; Wang Z. et al., 2022; Peng et al., 2023), in which they greatly promote the development of agricultural intelligence. Along with the arrival of the era of big data, the amount of image information increases exponentially, resulting in an increase in the amount of computation and training difficulty in the training process. This has also led researchers to pay more attention to lightweight networks, in order to maintain the accuracy of the premise of lightweight transformation of the network, MobileNet, ShuffleNet and other lightweight networks came into being, which can be better adapted to the evolving needs of the mobile market. To effectively alleviate the large amounts of computational resources and storage costs required for real-time image processing, Yuan et al. (2022) constructed a high-performance low-resolution MobileNet model, in which the network structure was simplified by cropping and the inception structure was used to fill the Dwise layer in a depth-separable convolution to extract the richer low-resolution features. The experimental results showed that the model achieved 89.38%, 71.60%, and 87.08% accuracies with the CIFAR - 10, CIFAR - 100, and CINIC - 10 datasets, respectively, and was suitable for real-time image classification tasks in low-resolution application scenarios. Fang et al. (2022) proposed a new network structure, HCA-MFFNet, for maize leaf disease recognition in complex contexts, and in order to validate the feasibility and effectiveness of the model in complex environments, it was compared with the existing methods, and the results proved that the model had an average recognition accuracy of 97.75% and an F1 value of 97.03%, which was the best overall performance. Hou et al. (2020) proposed a damage classification algorithm for castor seeds based on a convolutional neural network. Authors used castor seeds with missing shells or cracks and intact castor seeds to construct a dataset and build a network model to classify the seeds. The experimental results showed that the average accuracy was 87.78%, with 96.67% for castor seeds without shells, 80.00% for cracked castor seeds, and 86.67% for intact castor seeds; therefore, this method provided a feasible solution for the online real-time classification of castor seeds. Wang L. et al. (2022) designed a defect detection method based on the watershed algorithm and a two-channel convolutional neural network model, which can effectively identify defective and non-defective seeds with an average accuracy of 95.63%, an average recall of 95.29% and an F1 value of 95.46%. The assay provided an effective tool for the detection of corn seed defects. Cai et al. (2023) proposed a new grape leaf disease identification model, which was proved to have an identification accuracy of 93.26%, effectively providing decision-making information for the grape leaf disease identification system in precision agriculture. The above research results simplify the network structure complexity to a certain extent and reduced the requirement of hardware devices for model training. However, this is also often accompanied by poor model recognition accuracy, making it difficult to meet the needs of practical applications. Therefore, additional methods must be developed to improve the accuracy and achieve the purpose of accurate recognition.

In this study a seed-appearance quality assessment method based on an improved Inception-ResNet was proposed using intact and damaged maize seeds as test samples. By replacing depthwise separable convolution, adding an attention mechanism, and introducing a feature fusion strategy, the Inception-ResNet network structure was improved and optimized to obtain more detailed feature information, with the aims of achieving accurate, rapid, and nondestructive detections of seed appearance quality and providing a feasible reference scheme for subsequent automatic seed quality sorting processes.

## 2 Materials and methods

### 2.1 Experimental materials and treatment

#### 2.1.1 Image acquisition

**Figure 1:**
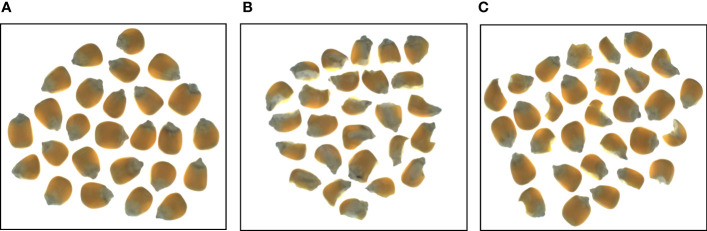
Corn seeds with different appearance qualities **(A)** Good seed grain, **(B)** Defective grain, **(C)** Both conditions.

**Figure 2:**
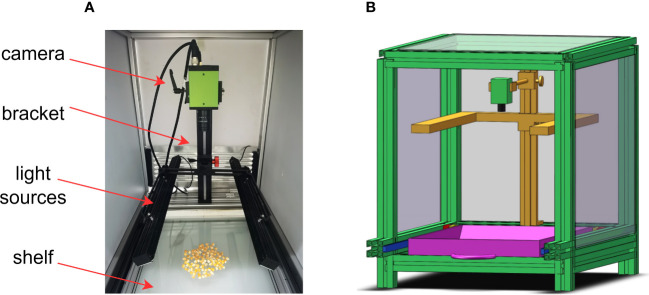
Image acquisition platform **(A)** physical device diagram and **(B)** 3D view of the device.

In this experiment, 50 groups of corn seeds with good appearance and broken appearance were collected respectively, with a total of 982 seeds, including 458 good seeds and 524 broken seeds. The training set and test set were divided according to 4:1. Ten sets of maize seed image data containing both good and defective seeds were used for verification of the final model (**Figure 1**). The image acquisition platform mainly consisted of four parts: a multispectral surface array camera from JAI Company in Denmark, with an image resolution of 2048×1536; a bracket to adjust the camera height; a light source on both sides of the camera; and a shelf to place the maize seed samples. The image data acquisition device is shown in **Figure 2**.

#### 2.1.2 Image processing

**Figure 3:**
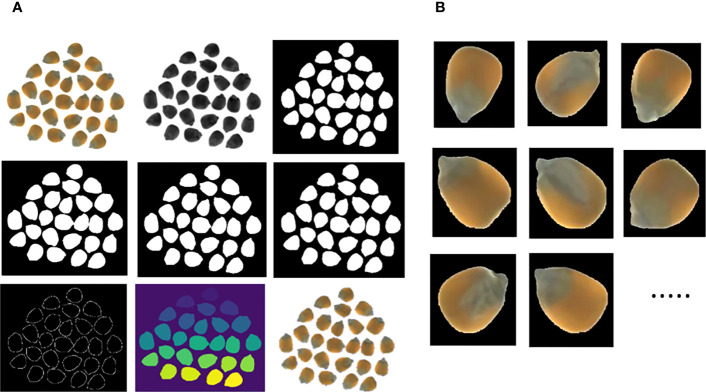
Process of watershed algorithm **(A)** Segmentation process and **(B)** Segmentation results.

The Python3.6 script language was used to segment the corn seed images, as shown in **Figure 3A**. First, the original color image was converted to grayscale, and a binary image was then obtained using the adaptive thresholding method, whereby the seed region is shown as white and the background region as black. White noise in the image was removed using a morphological open operation, and expansion was used in the foreground to distinguish the background and foreground areas of the image. The distanceTransform function was then used to obtain the center region of each seed, and the expanded image was subtracted from the central region to obtain the edge region. Finally, the watershed algorithm was used to mark the identified central region, delineate the seed boundary, and determine the range of each seed in the complete image by the location coordinates for segmentation. The segmentation effect is shown in **Figure 3B**.

### 2.2 Basic method and test environment

#### 2.2.1 Convolutional neural networks

**Figure 4:**
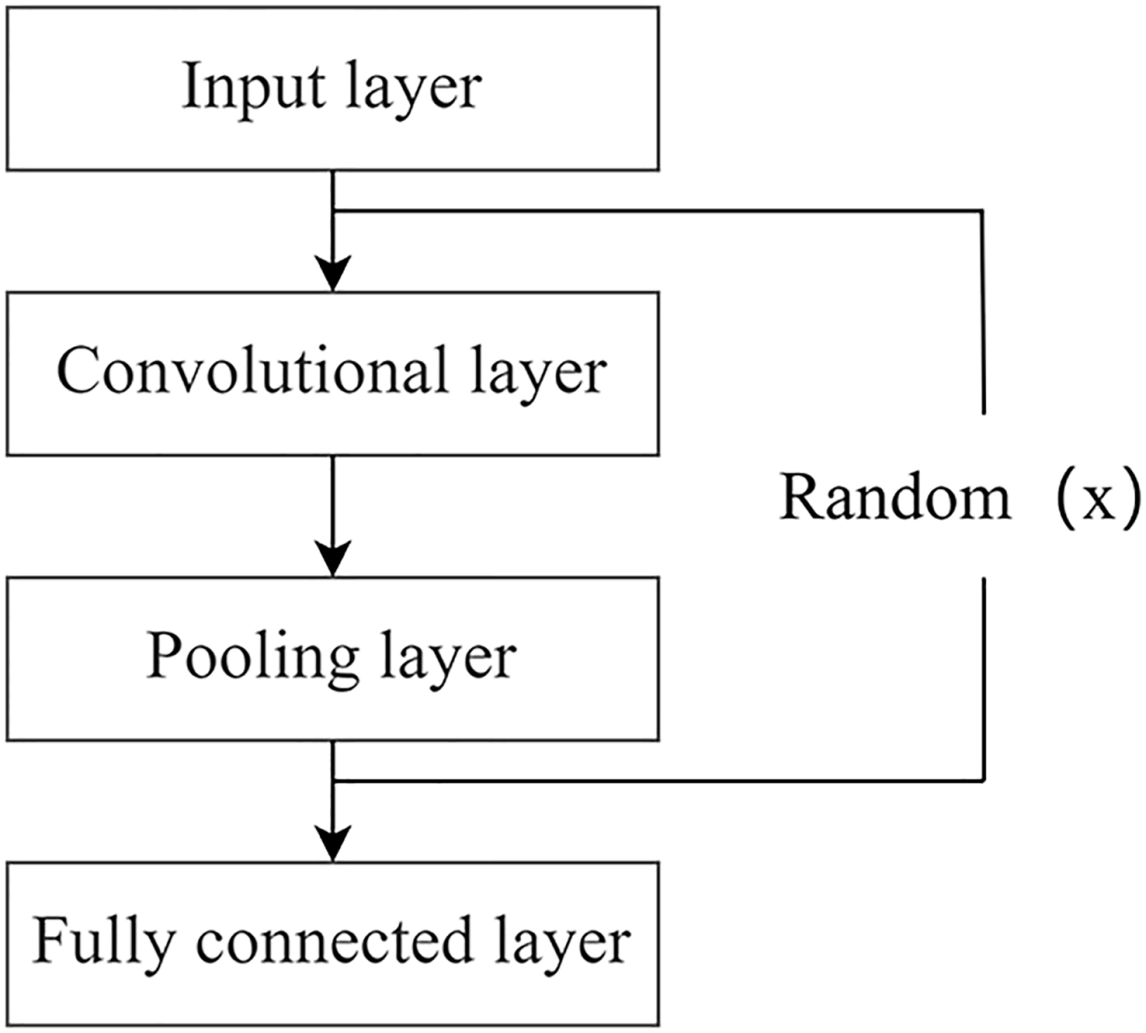
Basic structure of convolutional neural network.

A convolutional neural network (Rong et al., 2019) is a kind of multilayer perceptron. A traditional convolutional neural network consists of an input layer, convolutional layer, pooling layer, and fully connected layer. A simple neural network model can be formed by mixing different depths and stacking orders, as shown in **Figure 4**, in which the term random represents the number of times a particular layer of a structure is randomized.

The input layer represents the initial input of the entire convolutional neural network, and in the case of image processing, the input layer contains the pixel matrix of the input image, and the convolutional layer is the core component of convolutional neural network, which has the characteristics of local connection, weight sharing and translation invariance, and realizes the feature extraction function of the network. The pooling layer compresses the size of the input feature map while extracting the main features, thereby reducing the complexity of the network training process and the probability of overfitting the network model during this process. The fully connected layer is involved in weighting the features extracted from the convolutional and pooling layers, transforming them into layer vectors, and inputting one-dimensional data into the Softmax layer through multiplication operations to obtain the image classification results. It can be understood as a parallel, large-scale distributed processor that is capable of storing and using empirical knowledge.

#### 2.2.2 Depthwise separable convolution

**Figure 5:**
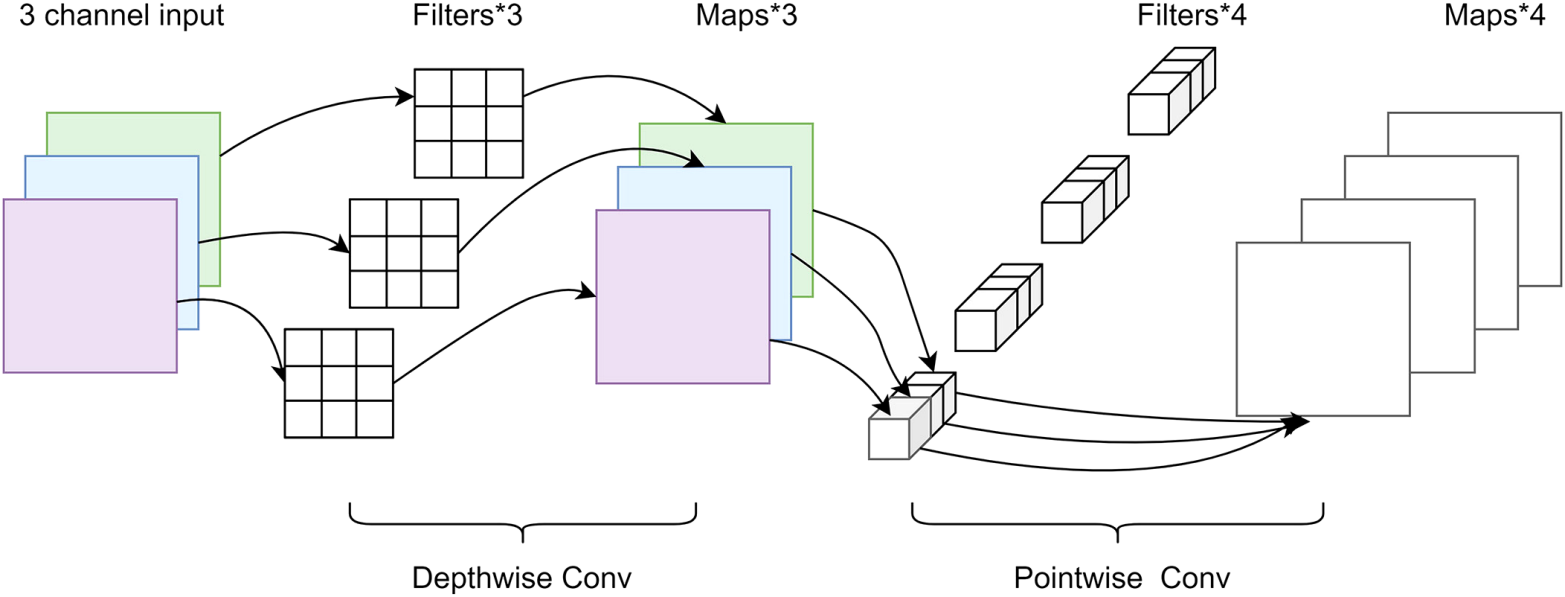
Depthwise separable convolution.

Depthwise separable convolution (Mamalet and Garcia, 2012) was proposed as a typical lightweight convolution structure that has a significantly reduced parameter number and increased training speed compared to a standard convolution and can separate channels and regions in the convolution operation. As shown in **Figure 5**, depthwise separable convolution mainly consists of two parts, depthwise convolution and pointwise convolution, which are used to extract feature information. One depthwise convolution kernel is responsible for one channel, and the number of feature maps generated by this process equals the number of input channels. The convolution operation is performed independently for each channel of the input layer, which does not make full use of the feature information from other channels in the same spatial location. Therefore, pointwise convolution is required to combine the feature maps to generate a new feature map, in which the number of convolution kernels corresponds to the output feature map.

Depthwise separable convolution effectively reduces the number of parameters required for the network model compared to the normal convolutional approach. The N H × W × C convolution kernels can be replaced by C H × W × 1 depthwise and N 1 × 1 × C pointwise convolution kernels. The number of depthwise convolution parameters is (H × W × 1) × C, and the number of pointwise convolution parameters is (1 × 1 × C) × N. The combined number of parameters for the depthwise separable convolution can be calculated as follows:


(1) Params=H×W×C+C×N

The number of parameters in the ordinary convolution is H × W × C × N, and the relationship between the two is compared as follows:


(2) $$\frac{H\times \text{W}\times \text{C}+\text{C}\times \text{N}}{H\times \text{W}\times \text{C}\times \text{N}}=\frac{1}{N}+\frac{1}{H\times \text{W}}$$

#### 2.2.3 Attention mechanism

The working principle of the attention mechanism is similar to the selective attention of human vision. It utilizes limited computational resources to focus on important feature information and emphasize regions of interest in a dynamically weighted manner, discarding irrelevant background information and nuisance information in the input features to improve network performance. In general, the attention mechanism involves the process of weight assignment, in which the input feature information is processed, the attention information is obtained through a weight assignment, and the attention mechanism is used to process these features. The process can be expressed as


(3) Attention=f(g(x),x)

Here, x represents the input feature information of the attention mechanism; g(x) represents the focus on the key areas, that is, the process of generating attention information by processing the input features; and f(g(x), x) represents the processing of the key areas, that is, processing of the input information using the attention information obtained from g(x).

#### 2.2.4 Evaluation indicators

In this study, objective evaluation criteria (Feng et al., 2022; Wang X. et al., 2022) were used to analyze the inspection model of the maize seed appearance quality using Accuracy(*A*), Precision (*P*), Recall (*R*), and by introducing the F1 value as the average evaluation of the reconciliation. The related formulae are as follows:


(4) $$A=\frac{\text{TP}+\text{TN}}{\text{TP}+\text{TN}+\text{FN}+\text{FP}}\times 100\text{\% }$$

(5) $$P=\frac{\text{TP}}{\text{TP}+\text{FP}}\times 100\%$$

(6) $$R=\frac{\text{TP}}{\text{TP}+\text{FN}}\times 100\%$$

(7) $$F1=\frac{2PR}{P+R}\times 100\%$$

#### 2.2.5 Test environment

We used a Windows 10, 64-bit operating system with a x64 based processor, Cuda version 11.0, and the Tensorflow deep learning framework based on the Python programming language. The computer contained an NVIDIA GeForce RTX 3090 graphics card with 24G video memory and a 12th Gen Intel(R) Core (TM) i7-12700KF processor at 3.61 GHz.

### 2.3 Algorithm improvement

#### 2.3.1 Improving the Inception-ResNet module

**Figure 6:**
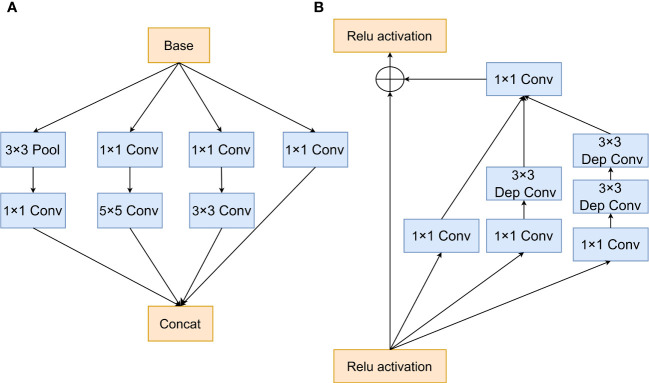
Inception module **(A)** Traditional Inception module and **(B)** Dinception-ResNet module.

The traditional Inception module, shown in **Figure 6A**, consists of 1 × 1, 3 × 3, and 5 × 5 convolution operations of various sizes for feature extraction using multiple-scale parallel convolution operations. The Inception-ResNet module (**Figure 6B**) combined the residual network structure of the ResNet with the separation of the large convolutional network into two tandem small convolutional structures to obtain the output feature maps of the 5 × 5 convolution, thereby improving the classification performance of the model. In this study, based on the Inception-ResNet module, the standard convolution part of the Inception-ResNet structure was replaced by a cost-effective convolution operation by combining the lightweight structure depthwise separable convolution (Dinception-ResNet). This approach could reduce the number of model parameters, increase the depth of the network, and enhance the feature extraction capability of the model, while preserving the feature diversity of the traditional inception multigroup structure.

A comparison of the parameters of the improved model is presented in **Table 1**. The original Inception-ResNet model used up to 28 979 618 parameters; this number was reduced by 177 920 after the depthwise separable convolutional replacement and the ratio of the number of trainable parameters to the total number of parameters was improved, which favors the design of lightweight networks.

**Table 1:** Parameter comparison.

Model	Total parameters	Trainable parameters	Non-trainable parameters
Inception-ResNet	28 979 618	28 947 106	32 512
Dinception-ResNet	28 801 698	28 770 338	31 360

#### 2.3.2 Adding attention mechanism

**Figure 7:**
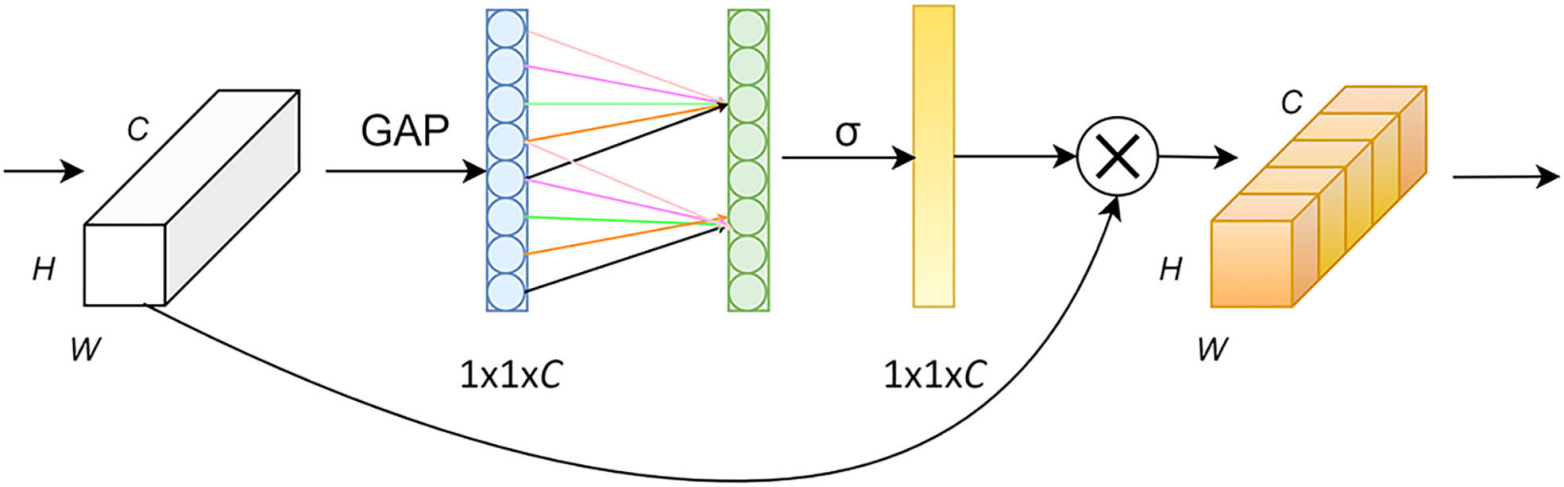
ECA network structure.

Introducing the Efficient Channel Attention Network after the Dinception-ResNet module effectively avoided dimensionality reduction and captured cross-channel interactions, as shown in **Figure 7**. The Efficient Channel Attention Network first transformed the input feature map from a matrix [H, W, C] into a vector [1, 1, C] using global averaging pooling, after which it calculated the adaptive 1D convolution kernel size based on the number of channels of the feature map, which was then used in 1D convolution. Subsequently, the weights of the feature maps with respect to each channel were obtained. Finally, the normalized weights and the original input feature maps were multiplied channel-by-channel to generate the weighted feature maps. These maps can be used to solve the information overload problem and improve the efficiency and accuracy of task processing by focusing on the more critical information for the current task, reducing the attention to other information, and using a small number of parameters to achieve suitable results. To fully demonstrate the effectiveness of Efficient Channel Attention Network, we conducted a comparison experiment between it and the commonly used attention mechanisms CBAM, SENet and CANet. By testing the relevant indexes of the experiments, the results were shown in **Table 2**, which clearly concluded that the overall detection performance of Efficient Channel Attention Network was optimal.

**Table 2:** Comparison of detection performance of different attention mechanisms.

Attention mechanism	Average Accuracy/%	Average Precision/%	Average Recall/%	F1/%	Detection time/s
ECANet	95.96	92.46	91.26	91.86	2.68
CBAM	89.68	91.33	89.68	90.50	2.65
SENet	83.33	87.34	83.33	85.29	2.67
CANet	90.48	91.89	90.47	91.17	2.66

#### 2.3.3 Introducing the feature fusion mechanism

**Figure 8:**
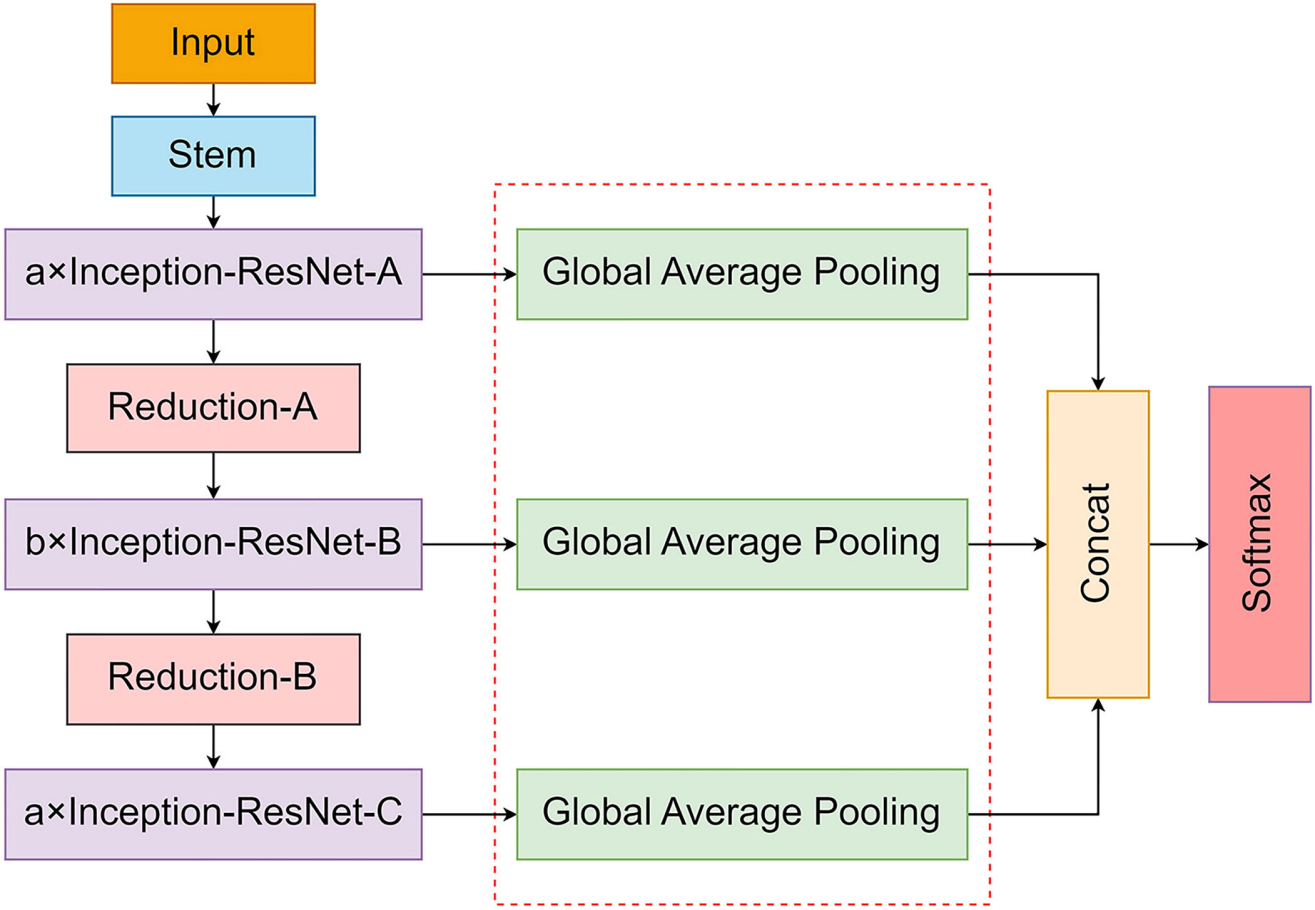
Network structure with added feature fusion.

As the depth of the network model increases, important feature information may be missed in the feature extraction process, thereby decreasing the classification accuracy of the model. Therefore, this study proposed feature fusion, as shown in **Figure 8**, in which the output features of each module were first pooled by global averaging to avoid overfitting. Subsequently, the output feature maps of the three stages were feature-fused to enrich the output feature information, enhance the generalization performance of the model, and improve the expression ability of the feature information.

#### 2.3.4 Improved network model structure

**Figure 9:**
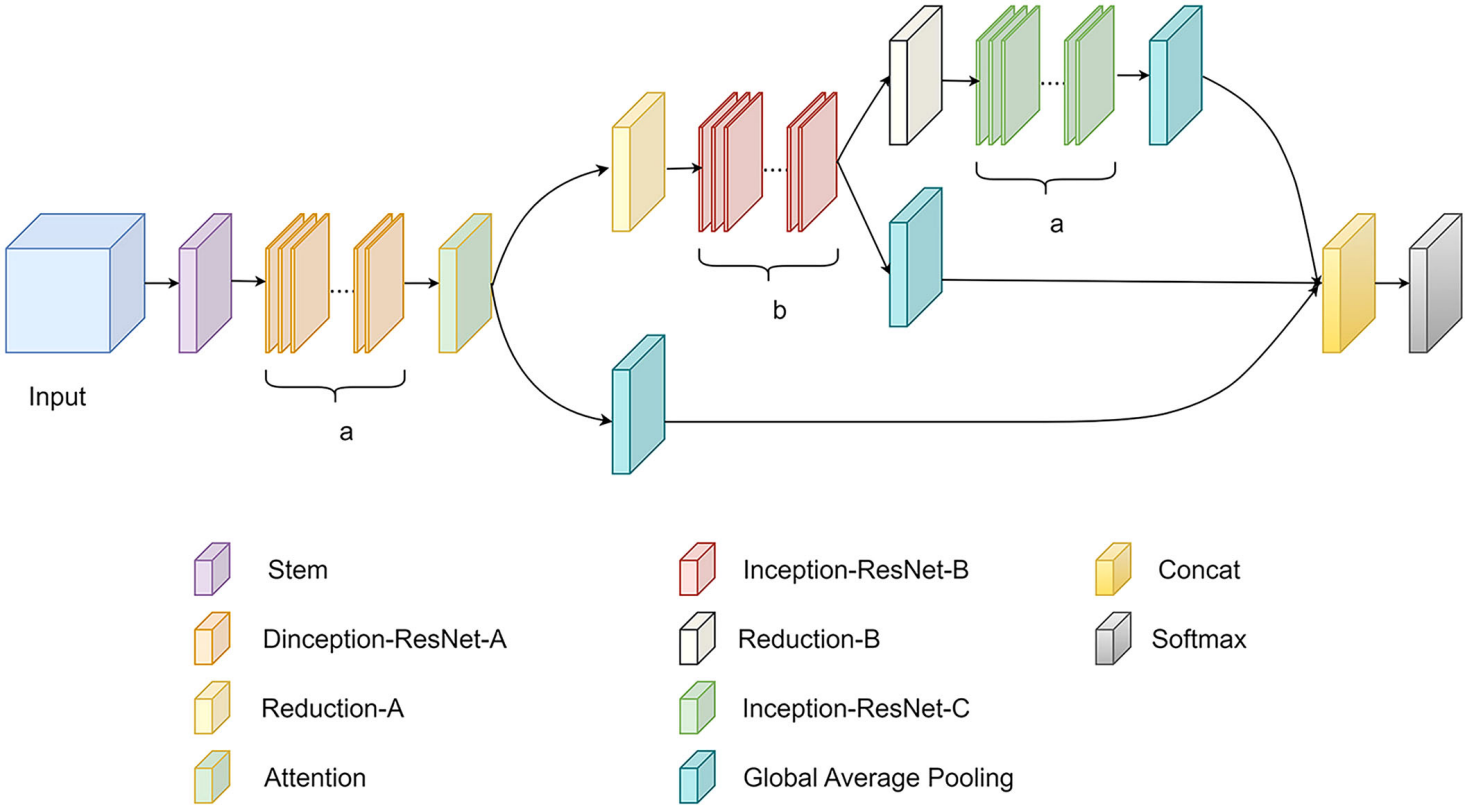
Improved model structure.

The traditional convolutional neural network structure generates a large number of parameters in the deep network. In addition, key features are mixed with irrelevant features, and the network is difficult to optimize; therefore, this study proposed an improved model based on Inception-ResNet (**Figure 9**). Depthwise separable convolution was used to replace the standard convolution in the Inception-ResNet module to reduce the number of model parameters, the network model was optimized by introducing the Efficient Channel Attention mechanism to increase the feature weights of key information and improve the network performance; in addition, the output features of the low, middle and high layers of the model were fused to improve the feature extraction ability of the model and enrich feature information to achieve the purpose of network optimization.

#### 2.3.5 Ablation study

In order to validate the effectiveness of the method proposed in this paper, separate experiments were conducted for the proposed modules to compare with the original Inception-Resnet model. By comparing the results of groups 1-2-3-4 in **Table 3**, it can be seen that the performance of each module reference is improved to some extent compared to the original network. Among them, the introduction of the feature fusion module resulted in the most obvious model performance improvement, with each performance index improved by 3.38 – 5.55 percentage points respectively. The average time for detecting an image was 2.62 seconds. Replacing standard convolution with depthwise separable convolution, the average time to detect an image was reduced by 30 milliseconds. By comparing the results of 1-2-5-6 groups and referring modules one by one, the model performance was improved and the effectiveness of the modules is fully proved.

**Table 3:** Comparison of improving module performance.

Model		Average Accuracy/%	Average Precision/%	Average Recall/%	F1/%	Detection time/s
1	Inception-Resnet	88.10	90.26	88.10	89.17	2.59
2	Inception-Resnet (+depthwise separable convolution)	89.68	91.33	89.67	90.49	2.56
3	Inception-Resnet (+ECANet)	95.96	92.46	91.26	91.86	2.68
4	Inception-Resnet (+feature fusion)	93.65	94.28	93.65	93.96	2.62
5	Inception-Resnet (+depthwise separable convolution + ECANet)	92.06	93.05	92.06	92.55	2.77
6	Improved Inception-ResNet	96.03	96.27	96.03	96.15	2.44

## 3 Results and analysis

### 3.1 Model optimization

**Figure 10:**
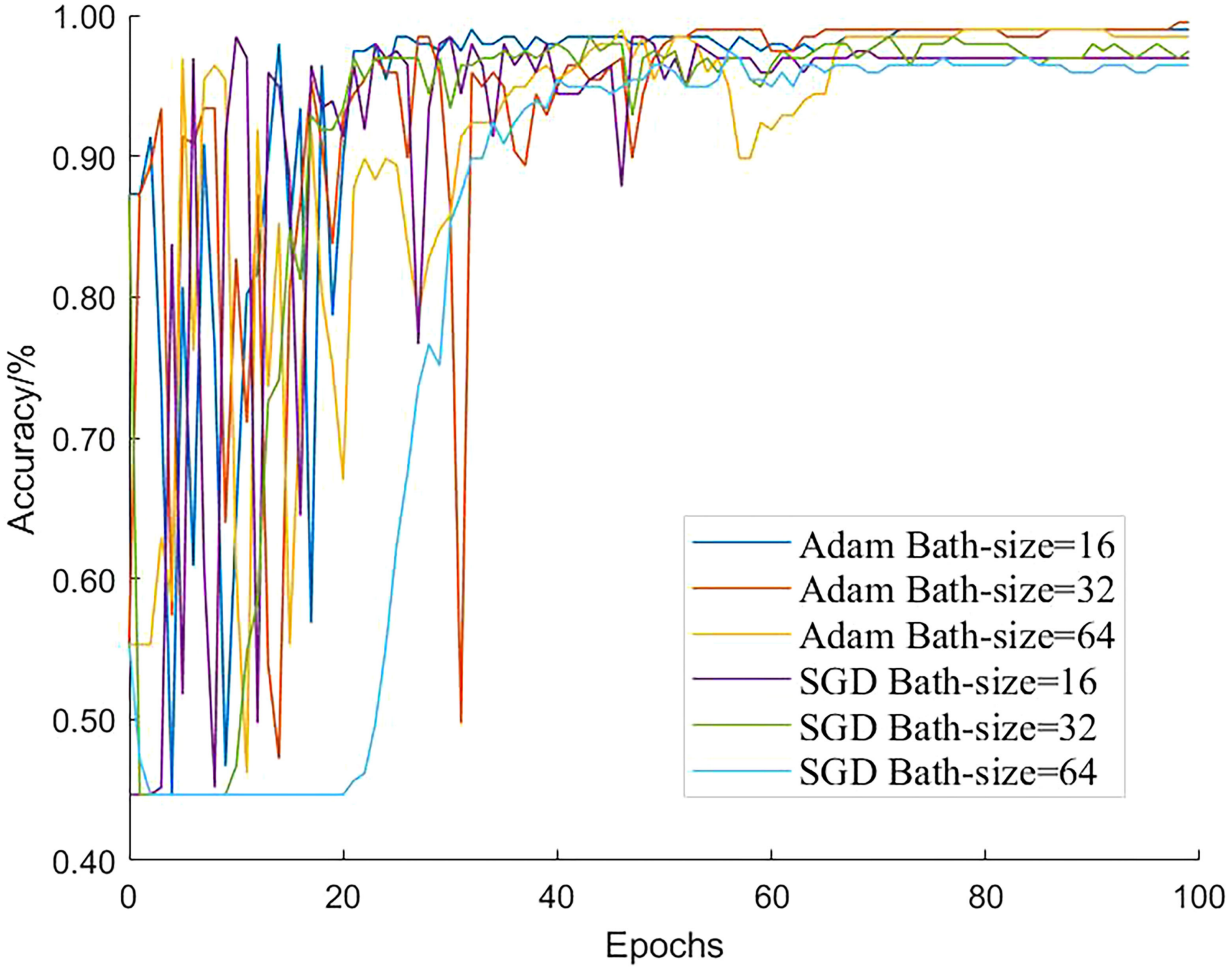
Comparison of parameters.

Different networks apply different model parameters. By adjusting certain parameters in the model and using test set recognition accuracy as the evaluation index, we investigated the effects of the parameters on the classification accuracy of the model. In the convolutional neural network model, batch size is an important hyperparameter, and we chose a batch size that was appropriate to train the model to converge to the global optimum. Using a large number of parameters in a deep convolutional neural network creates correction issues; therefore, a suitable optimizer was selected to improve the model training speed and accuracy. The training parameter batch sizes of the model were 16, 32, and 64, and two different optimization algorithms, Adam and SGD, were used. The number of model iterations was set to 100 and the learning rate was 0.001; the final test set accuracy variation curve is shown in **Figure 10**. These settings ensured that when the Adam optimizer was selected for the improved model and the bath size was set to 32, the accuracy of the test set was the highest and the detection performance was optimal.

### 3.2 Comparison of machine learning experiments

**Figure 11:**
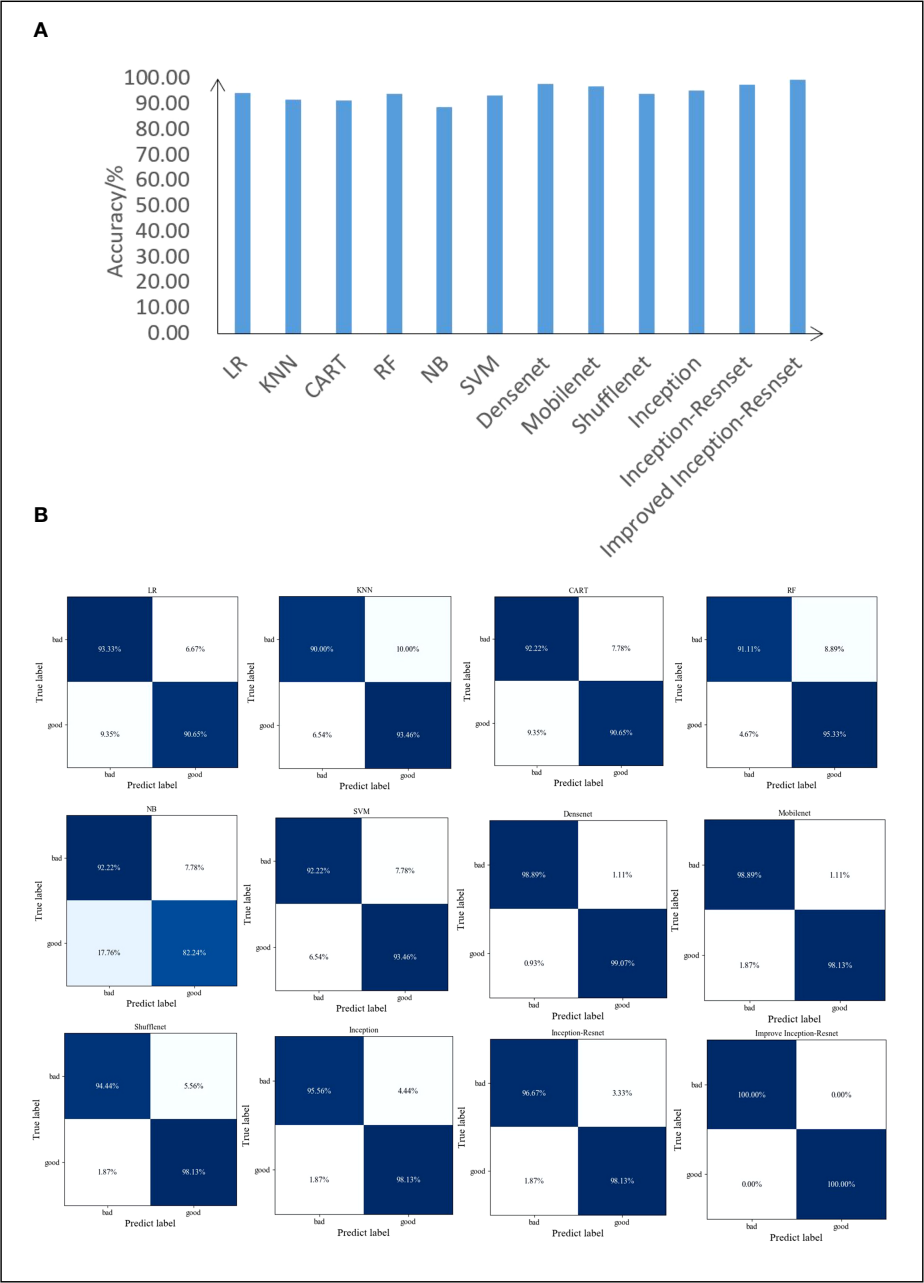
Test result **(A)** Comparison of accuracy between different model test sets and **(B)** Confusion matrix of different models.

To verify the performance of the improved model, three features of maize seed (shape, color, and texture) were extracted using traditional machine vision techniques, and the three types were combined to quantify the images more effectively. The logistic regression, k-nearest neighbor, decision tree, random forest, gaussian naïve bayes, and support vector machine models were used for classification, and the common lightweight convolutional neural networks DenseNet, MobileNet, ShuffleNet, and Inception, and the original Inception-ResNet were selected for comparison. The dataset was divided into training and test sets in a 4:1 ratio, and the classification accuracies of the different models on the test set are shown in **Figure 11A**. The highest accuracy with the test set occurred with the improved Inception-ResNet model, with 99.49%, which was an increase of 2.03 percentage points over the accuracy of the original model of 97.46% and an increase of 5.07 percentage points compared to the LR model, which had the highest machine-learning recognition accuracy for the test set of 94.42%. In addition, a confusion matrix was chosen as the visual presentation tool to evaluate the quality of the classification models, and the matrices for several models of the test set are shown in **Figure 11B**. The plots showed that the original Inception-ResNet model had an accuracy of 96.67% for the identification of seeds with a defective appearance and 98.13% for the identification of seeds with a good appearance. The confusion matrix for the improved Inception-Resnet model showed the best results, with a significant improvement and a classification accuracy of the test set seeds of up to 100%; therefore, this model provided an accurate identification of seed appearance quality.

### 3.3 Comprehensive testing performance

To evaluate the effectiveness of the improved Inception-ResNet in detecting seed appearance quality, ten sets of image data with good and bad co-existing seeds were selected for the final validation and performance of the six detection algorithms, DenseNet, MobileNet, ShuffleNet, Inception, Inception-ResNet, and improved Inception-ResNet. The models were evaluated using the criteria of average accuracy, precision, recall and the reconciled average evaluation F1 value. The results are shown in **Table 4**, and the highest average accuracy of Inception-ResNet after improvement reached 96.03%, which was 3.23 – 11.11 percentage points higher than those of the other models, the average precision reached 96.27%, an improvement of 3.46 – 8.00 percentage points compared to other networks. Similarly, the average recall was 3.22 – 11.11 percentage points greater than the other models, with 96.03%, while the reconciled average evaluation F1 value reached 96.15%, which was 3.34 – 9.59 percentage points higher than the other algorithms and the average time to achieve real-time detection was 2.44 seconds per detected image.

**Table 4:** Comparison of detection model performances.

Model	Average Accuracy/%	Average Precision/%	Average Recall/%	F1/%	Detection time/s
DenseNet	84.92	88.27	84.92	86.56	2.86
MobileNet	87.30	89.75	87.30	88.51	1.81
ShuffleNet	88.87	90.24	88.88	89.55	1.91
Inception	92.80	92.81	92.81	92.81	2.55
Inception-ResNet	88.10	90.26	88.10	89.17	2.59
Improved Inception-ResNet	96.03	96.27	96.03	96.15	2.44

### 3.4 Model performance

**Figure 12:**
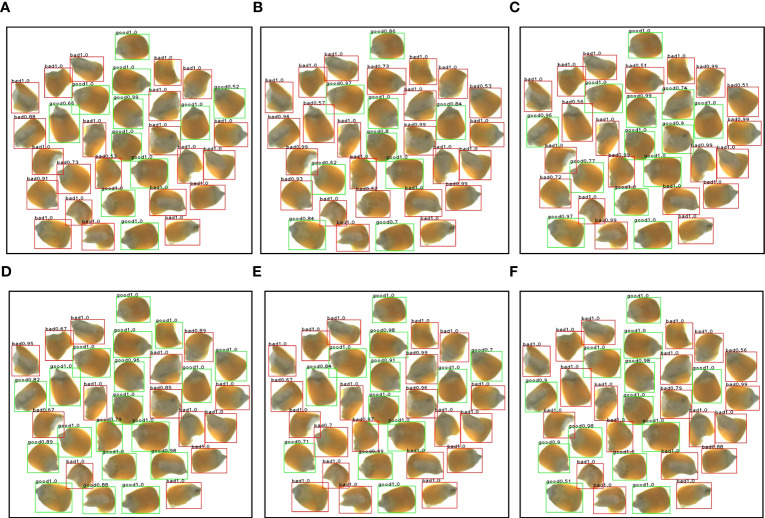
Comparison of detection results of multiple algorithms **(A)** DenseNet **(B)** MobileNet **(C)** ShuffleNet **(D)** Inception **(E)** Inception-ResNet **(F)** Improved Inception-ResNet.

Combined with the watershed algorithm to obtain the specific location coordinates of the corn seeds for defect detection, the detection results of several models are shown in **Figure 12**. The improved Inception-ResNet model had a significantly greater number of cases with target confidence levels of 1.0, with approximately 88% of the seed identifications showing a level approaching 1.0, which is a six-percentage point improvement over the level of the original model. A comparison of the detection results is shown in **Table 5**, which shows that the improved Inception-ResNet model had the greatest number of correct detections and highest overall recognition accuracy, thereby allowing for an accurate detection of the appearance quality of corn seeds to achieve the desired results. In the actual detection process, there are also cases of misdetection, such as the possibility of detecting bad seed as good seed when the area of damage is small. When the seeds are randomly placed at an inappropriate Angle or there is serious adhesion leading to area coverage between seeds, it is possible to detect good seeds as bad seeds. This also indicates that there is room for model refinement and we need to follow up to explore more accurate detection models.

**Table 5:** Comparison of test results.

Model	Correct number	Miss number
DenseNet	27	6
MobileNet	25	8
ShuffleNet	25	8
Inception	27	6
Inception-ResNet	28	5
Improved Inception-ResNet	29	4

## 4 Discussion

We proposed an improved model based on Inception-Resnet for corn seed appearance quality detection, which can realize the purpose of accurate and real-time detection. However, the model is domain dependent and mainly focuses on the detection of seed appearance quality, which makes it difficult to directly migrate the network to other domains. This is mainly due to the fact that the network structure modifications are performed based on seed features, which are difficult to guarantee that they will respond well on datasets from other domains. With the development of agricultural digitization, it can be applied to seed intelligent sorting equipment to guarantee the quality of seeds. We can do further research to solve some problems, firstly, the data collection was conducted indoors, in the future, we can try to train the model directly for the dataset collected in the actual outdoor environment to improve the generalization ability of the model; secondly, we can design a lighter network structure, which can ensure the stability of the algorithm and efficiently deploy it on different platforms while pursuing high performance; finally, we only study the appearance damage of corn seeds, and in the future we can continue to explore more specific defects on the seed surface, such as diseases and pests, and further promote the development of the seed industry.

## 5 Conclusions

To improve the accuracy of seed appearance quality assessment, this study proposed an improved Inception-ResNet model based on the Inception-ResNet algorithm for identifying appearance defects of corn seeds and obtained the following conclusions: (1) Taking advantage of the small number of depthwise separable convolutional parameters as opposed to the standard convolution in the Inception-ResNet module reduced the large number of parameters generated by overlaying the Inception-ResNet module and the requirement for hardware resources. (2) Introducing the Efficient Channel Attention Network strengthened the ability to learn key information and avoided the problem of excessive information storage and information overload in the model. Simultaneously, the output feature maps were fused to obtain richer feature information to enhance the network generalization ability and improve network performance. (3) The detection effect of the method proposed in this study was superior to the other models tested, with an average accuracy of 96.03%, average precision of 96.27%, average recall of 96.03%, F1 value of 96.15%, and detection speed for a single corn seed image of approximately 2.44 seconds. The performance index improved significantly with high performance stability, providing a theoretical basis for subsequent seed quality detection.

## Data availability statement

The raw data supporting the conclusions of this article will be made available by the authors, without undue reservation.

## Author contributions

CS, BP, and XF conceived the idea, proposed the method. HW and YZ set up the platform and collected data. CS and BP write the code, test the results and write the manuscript. LS and XS contributed to the validation results. XF and XS provided guidance for the writing of this paper. All authors contributed to the article and approved the submitted version.
